# The Role of Dimorphism Regulating Histidine Kinase (Drk1) in the Pathogenic Fungus *Paracoccidioides brasiliensis* Cell Wall

**DOI:** 10.3390/jof7121014

**Published:** 2021-11-26

**Authors:** Marina Valente Navarro, Yasmin Nascimento de Barros, Wilson Dias Segura, Alison Felipe Alencar Chaves, Grasielle Pereira Jannuzzi, Karen Spadari Ferreira, Patrícia Xander, Wagner Luiz Batista

**Affiliations:** 1 Department of Microbiology, Immunology and Parasitology, Federal University of São Paulo, São Paulo 04023-062, Brazil; marinavnavarro@hotmail.com; 2 Department of Pharmaceutical Sciences, Federal University of São Paulo, Diadema 09913-030, Brazil; barros_yasmin@hotmail.com (Y.N.d.B.); segurawd@gmail.com (W.D.S.); karen.spdari@unifesp.br (K.S.F.); patricia.xander@unifesp.br (P.X.); 3 Center of Toxins, Immune-Response and Cell Signaling—CeTICS, Instituto Butantan, São Paulo 05503-900, Brazil; felipealison@gmail.com; 4 Department of Clinical and Toxicological Analyses, University of São Paulo, São Paulo 05508-000, Brazil; grasi_jannuzzi@hotmail.com

**Keywords:** histidine kinase, dimorphism, *Paracoccidioides*, paracoccidioidomycosis, cell wall

## Abstract

Dimorphic fungi of the *Paracoccidioides* genus are the causative agents of paracoccidioidomycosis (PCM), an endemic disease in Latin America with a high incidence in Brazil. This pathogen presents as infective mycelium at 25 °C in the soil, reverting to its pathogenic form when inhaled by the mammalian host (37 °C). Among these dimorphic fungal species, dimorphism regulating histidine kinase (Drk1) plays an essential role in the morphological transition. These kinases are present in bacteria and fungi but absent in mammalian cells and are important virulence and cellular survival regulators. Hence, the purpose of this study was to investigate the role of PbDrk1 in the cell wall modulation of *P. brasiliensis*. We observed that PbDrk1 participates in fungal resistance to different cell wall-disturbing agents by reducing viability after treatment with iDrk1. To verify the role of *PbDRK1* in cell wall morphogenesis, qPCR results showed that samples previously exposed to iDrk1 presented higher expression levels of several genes related to cell wall modulation. One of them was *FKS1*, a β-glucan synthase that showed a 3.6-fold increase. Furthermore, confocal microscopy analysis and flow cytometry showed higher β-glucan exposure on the cell surface of *P. brasiliensis* after incubation with iDrk1. Accordingly, through phagocytosis assays, a significantly higher phagocytic index was observed in yeasts treated with iDrk1 than the control group, demonstrating the role of PbDrk1 in cell wall modulation, which then becomes a relevant target to be investigated. In parallel, the immune response profile showed increased levels of proinflammatory cytokines. Finally, our data strongly suggest that PbDrk1 modulates cell wall component expression, among which we can identify β-glucan. Understanding this signalling pathway may be of great value for identifying targets of antifungal molecular activity since HKs are not present in mammals.

## 1. Introduction

Paracoccidioidomycosis (PCM) is a systemic granulomatous human disease endemic in Latin America. It is caused by *Paracoccidioides* spp., a thermally-dimorphic fungus that presents as an infective mycelium in the environment, and it switches to a pathogenic yeast form in the mammalian host [1,2]. Its clinical manifestations occur in two distinct forms, acute or subacute and chronic [3], affecting mainly the lungs, but it is capable of spreading to other tissues [4,5]. Primary infection usually occurs by inhaling propagules (conidia) produced during the mycelial form [6]. Once inhaled, fungal propagules will be recognized by cells of the innate immune system [7]. The recognition of fungal cell wall components begins with pathogen-associated molecular patterns (PAMPs) through pathogen recognition receptors (PRRs). These receptors include Toll-like receptors (TLRs), mannose receptors, complement pathway molecules and lectin family receptors (CLRs), such as dectin-1 [8]. The interaction of these molecules with fungal yeasts leads to activation of the innate immune response, consequently activating mediators involved in eliminating these pathogens and controlling the adaptive immune response [9].

However, fungi have mechanisms to prevent their elimination by the host’s immune system [10]. Several fungi that engage in morphological transitions are of great medical importance, such as *Talaromyces marneffei* (*Penicillium marneffei*), *Blastomyces dermatitidis*, *Coccidioides immitis*, *Histoplasma capsulatum*, *Sporothrix schenckii*, and *Paracoccidioides* spp. [11]. The transition in *Paracoccidioides* spp. and other dimorphic fungi is essential for the establishment of the disease [12]. This alters not only the cell morphology but also the composition of the cell wall elements. In *Paracoccidioides* spp. mycelium, there is a prevalence of β-1,3-glucan and β-1,6-glucan, and in the yeast form, there is a prevalence of α-1,3-glucan and chitin [13]. This ensures fungal survival in the host environment since the content of α-1,3-glucan is correlated with the degree of fungal virulence [14]. In addition, masking the presence of β-1,3-glucan molecules, a highly immunogenic structure, is recognized by the dectin-1 receptor of phagocytic cells [15]. The morphological switch is believed to be an additional evasion strategy against phagocytic cells and mechanisms for recognition of the cell wall components [15]. In this context, the fungal cell wall plays an important role in immunological recognition.

In *Paracoccidioides* spp., different genes are expressed according to the phase (yeast or mycelium) [16]. The mechanism of this transition has been unclear. However, genes related to the control of the mycelium-yeast transition (M-Y) have recently been identified in *B. dermatitidis* and *H. capsulatum*, including dimorphism-regulating histidine kinase (*DRK1*). *DRK1* is mainly expressed in the yeast phase [11] of *B. dermatitidis* [17], *S. schenckii* [18], and *T. marneffei* [19] and, more recently, it was characterized in *P. brasiliensis* [20].

Histidine kinases (HKs) were discovered in the 1980s in *Escherichia coli* [21], and were believed to be present only in bacteria. In the 1990s, they were also discovered in plants, fungi, archaea, cyanobacteria and amoebas [22]. In fungi, the functions attributed to HKs have not been explored very well [23]. HKs are classified based on phylogenetic analyses; in fungi, there are 16 groups, determined by the C- and N-terminal regions and the domains present in each group [22,24,25]. This is a signal transduction mechanism that contains a conserved kinase domain and a conserved regulatory domain. After an extracellular stimulus, the HK domain is autophosphorylated on a histidine residue, followed by a phosphate group transfer to the regulatory domain in an aspartate residue, which catalyses a downstream reaction of the effector domain that leads to downstream signalling [26].

Among the characterized pathways in fungi, we can mention the response to osmotic stress [25], oxidative protection against phagocytic cells [27] and regulation of the dimorphism in pathogenic fungi [17,18,20,28]. In *P. brasiliensis*, *DRK1* is known to be a group III histidine kinase essential to the dimorphic transition process [20].

Since HKs are known to regulate morphological switches in *P. brasiliensis*, this work aimed to characterize the PbDrk1 protein, which is involved in the transition from mycelium to yeast. This investigation is of great interest since HKs use a phosphorylation mechanism where the amino acid phosphoryl-receiving groups are aspartate and histidine residues, unlike the serine, threonine and tyrosine residues that are prevalent in mammals. Thus, knowing that these molecular sensors are absent in humans, it is extremely important to study the components that are part of these activation pathways, as they may represent potential molecular targets in the development of new antifungal agents.

## 2. Materials and Methods

### 2.1. Fungal Isolates and Growth Conditions

The *P. brasiliensis* isolate Pb18 was grown in yeast peptone dextrose modified medium (mYPD) (0.5% yeast extract, 1% peptone, and 0.5% glucose, pH 6.7) for 4 to 5 days at 37 °C and shaking at 150 rpm. For mycelium growth, viable yeast cells were cultivated in mYPD at 25 °C for 7 days at 150 rpm. Viability was assessed by Trypan blue 0.4% counting on Neubauer’s chamber, using the formula: cell viability(%) = viable cells /total cells × 100. All chemicals were purchased from Sigma-Aldrich (St. Louis, MO, USA) unless otherwise mentioned.

### 2.2. Histidine Kinase Inhibitor Susceptibility

About 1 × 10^6^ yeast were incubated with different concentrations (100, 50, 25, 12.5, and 6.25 µg/mL) of Fludioxonil (Thermo Scientific, Waltham, MA, USA), a specific inhibitor of class III histidine kinase (iDrk1). The inhibitor was solubilized in DMSO (dimethylsulfoxide). Yeasts were incubated for 24 h under constant agitation of 180 rpm at 37 °C. Each yeast culture was diluted (10, 50, 100, 500, and 1000 times) in YPDmod broth, and 10 μL of each suspension was plated to YPDmod agar medium. Plates were photographed after 7 days of growth at 37 °C. This assay was performed in biological triplicate.

### 2.3. Dimorphic Transition Assay

Yeast cells of Pb18 were grown in mYPD agar (pH 6.5) at 37 °C for 4 to 5 days and inoculated in mYPD broth medium (pH 6.5). Then yeasts were incubated at 25 °C for five to six days to reverse yeast to mycelium entirely. After the complete transition, yeasts were centrifuged at 3000× *g* and washed with PBS buffer (pH 7.2). The mycelium was then seeded in 6-well plates and 20 µM iDrk1 (Fludioxonil) was added. The samples were monitored every 24 h under an optical microscope (Zeiss) at 100× magnification. Every 24 h, the culture medium was supplemented with 20 µM fludioxonil (because of inhibitor photodegradation). With each addition, a new solution was prepared to guarantee its activity. This assay was performed in a biological duplicate.

### 2.4. Cell Wall Disturbing Agents Spot Test

The sensitivity of Pb18 to cell wall disruptors was investigated using the spot assay. About 1 × 10^6^ yeasts were incubated with iDrk1 (25 µg/mL) for 24 h at 37 °C at 150 rpm. Each yeast culture was diluted (10, 50, 100, and 500 times) in YPDmod broth, and 10 μL of each suspension was applied to mYPD agar medium supplemented with different cell wall disrupting agents, such as: Congo Red (Congo Red) (2.5 µM), Calcofluor White (1 µg/mL) and sodium chloride (150 mM). The plates were incubated for seven days at 37 °C, and then photographed. This assay was performed in biological triplicate.

### 2.5. RNA Extraction and Real-Time Quantitative PCR Analysis

Pb18 yeasts were grown for four to five days in YPDmod pH 6.5 medium at 37 °C and 150 rpm, counted and the volume was adjusted to 30 mL at a concentration of 1 × 10^6^ cells/mL. Then, yeasts were incubated with 25 µg/mL of iDrk1 for 24 h at 37 °C. After this incubation period, RNA extraction was performed. Samples were centrifuged at 3000× *g* for 10 min at 4 °C and washed 3 times with PBS (pH 7.2). Then, in 15 mL tubes, approximately 500 µL of glass beads (425–600 µm—Sigma-Aldrich, San Louis, MO, USA) and 1.5 mL of TRizol^®^ (Invitrogen, Waltham, MA, USA) were added to the sample. The tubes were vigorously vortexed for 6 cycles of 1 min, alternating with 1 min on ice. RNA extraction was performed as previously described [29]. Quantification was performed using spectrophotometry (NanoDrop 2000/2000c, Thermo Fisher Scientific, Waltham, MA, USA). For complementary DNA (cDNA) synthesis, 500 ng of RNA was initially submitted to the DNase I enzyme (Thermo Fisher Scientific, Waltham, MA, USA) and then to ProtoScript First Strand cDNA Synthesis kit (New England BioLabs, Ipswich, MA, USA), according to the manufacturer’s instructions. To assess gene expression by real-time quantitative PCR, the reaction was performed with SYBR^®^ Green Master Mix (Applied Biosystems, Foster City, CA, USA) according to the manufacturer’s instructions. The endogenous expression genes for ribosomal protein 60S L34 (L34r) and 18S were used as normalizing controls. For each gene of interest and normalizer gene, a negative reaction control was also added. The samples were prepared in triplicate to a 96-well plate (0.2 mL MicroAmp™ Optical 96-Well Reaction Plate—Applied Biosystems) compatible with the equipment used, and the plate was sealed with an optical adhesive (MicroAmp™ Optical Adhesive Film—Thermo Fisher Scientific, Waltham, MA, USA). The equipment used was the ABI StepOne Plus Real-Time PCR System (Applied Biosystems) with the following conditions: 10 min at 95 °C, followed by 40 cycles of 15 s at 95 °C and 1 min at 60 °C. The dissociation curve included an additional cycle of 15 s at 95 °C, 20 s at 60 °C, and 15 s at 95 °C. The curves of oligonucleotides efficiency were evaluated from a cDNA obtained previously and serially diluted (100, 10, 1, and 0.1 ng/μL). The Ct values of each dilution point were determined and used to make the standard curve and finally calculate the primer efficiency (E = 10^(−1⁄slope)−1^ × 100). The relative expression was determined based on the 2^−ΔΔCt^ method [30]. The sequences used for each gene are listed in Table 1.

### 2.6. Quantification of Cell Wall Components

About 1 × 10^6^ yeast cells were grown and incubated for 24 h with 25 µg/mL of iDrk1 in mYPD at 37 °C under stirring at 180 rpm. Then, yeasts were collected, homogenized in blocking solution (0.5% BSA, 5% rabbit serum, 5 mM EDTA, 2 mM NaN_3_ in PBS, pH 7.2) and incubated for 30 min at room temperature. Then, yeasts were incubated with 1 µg/mL of the β-glucan binding probe for 1 h on ice. This probe corresponds to the human Dectin-1 receptor fused to the FC portion of mouse IgG1 (Sino Biological, Beijing, China). Samples were incubated with Alexa-488-conjugated anti-mouse IgG secondary antibody (Molecular Probes, Eugene, OR, USA) at a 1:200 ratio for 45 min on ice. Between each incubation, step cells were washed three times with wash buffer (0.5% BSA, 5 mM EDTA, 2 mM NaN_3_ in PBS, pH 7.2). Determination of chitin oligomers was performed with the WGA (wheat germ agglutinin) marker conjugated to FITC (Sigma-Aldrich, San Louis, MO, USA), at a concentration of 25 µg/mL in 1 mL of PBS (pH 7.2). Mannan determination was performed with FITC-conjugated Concanavalin A (Sigma-Aldrich, San Louis, MO, USA), at a concentration of 25 µg/mL in 500 µL of PBS (pH 7.2). Both samples were incubated for 1 h protected from light under agitation at 800 rpm. Next, samples were centrifuged at 3000× *g* for 5 min at 4 °C and washed three times with 1 mL of PBS (pH 7.2). Finally, all samples were homogenized in 500 µL of PBS (pH 7.2) and analyzed by flow cytometry (BD FACSCaliburTM, Becton Dickinson) using the FL-1 detection channel. Each sample was prepared in experimental triplicate. A total of 10,000 events were counted and quantification graphs were generated from the median fluorescence intensity (MFI). The data obtained were analyzed using FlowJo software version 10.6.2 (FlowJo, LLC, FlowJo™, Ashland, OR, USA) [31].

### 2.7. Confocal Microscopy

To evaluate the exposion of β-glucan molecules through fluorescence confocal microscopy, cells were prepared as described on the previous section and then centrifuged and homogenized in PBS:glycerol (3:1). The slides were prepared with 30 µL of the sample, sealed and analyzed under a confocal microscope (SP8 Lightning, Leica Microsystems). Fluorescence intensity was quantified using ImageJ analysis software (version 1.53i) through corrected total cell fluorescence (CTCF).

### 2.8. PKA Activity and cAMP Quantification

PKA activity was performed using the PKA Colorimetric Activity Kit (Thermo Fisher Scientific, Waltham, MA, USA), according to the manufacturer’s specifications. For this purpose, 1 × 10^7^ Pb18 cells were incubated with or without 25 µg/mL of iDrk1 or 1 mM H_2_O_2_ for 30 min in YPDmod at 37 °C and 180 rpm. Yeasts were collected and washed with PBS (pH 7.2), centrifuged for 3000× *g* for 10 min and homogenized in Tris-based lysis buffer contained in the kit. Lysis was assessed by adding glass beads and vigorous vortexing (5 cycles of 1 min interspersed with 1 min of incubation on ice). Samples were then centrifuged and the supernatant was recovered. For PKA activity assay, 1 mg of protein was used. Intracellular levels of cyclic AMP (cAMP) were quantified by the Cyclic AMP ELISA Kit (Cayman Chemical, Ann Harbor, MI, USA). Pb18 yeasts were prepared as described above. Cellular lysis was performed according to the manufacturer’s specifications, using 10 mM HCl and glass beads. For cAMP quantification, it was used about 0.5 μg of protein. The total protein extracts quantification was performed by Bradford assay [32]. Absorbance was read at 405 nm (BioTek—Synergy HT) and data were plotted in triplicate from the standard curves absorbance values.

### 2.9. Glycogen Accumulation

A 10 mL suspension of 5 × 10^6^ Pb18 yeasts/mL was incubated with or without 25 µg/mL of iDrk1 for 24 or 48 h, followed by centrifugation at 2500 rpm for 3 min (the supernatant was discarded). The pellet was homogenized in 1 mL of iodine solution (0.2% iodine and 0.4% potassium iodide) and incubated for 3 min at room temperature. The samples were again centrifuged at 2500 rpm for 3 min, the supernatant was discarded and the pellet homogenized in 30 μL of the iodine solution. The samples were plated to a 96-well plate, 200 µL of PBS (pH 7.2) was added to each well and the samples were photographed [33].

### 2.10. Phagocytosis Assay

In vitro phagocytosis was performed with the J774 macrophage cell line. About 2.5 × 10^5^ viable cells were plated in a 24-well plate containing RPMI (Gibco, Gaithersburg, MD, USA) supplemented with 10% FBS. A 15 mm diameter circular sterilized coverslip was added to each well. After adhesion, macrophages were primed with 100 ng/mL of LPS 30 min before interaction with *P. brasiliensis*. J774 cell line is activated by LPS showing changes in morphology, such as cytoplasm expansion, contributing to a better performance in phagocytosis assays [34,35]. The interaction was carried out in a 2:1 ratio (yeast:macrophages) and incubated for 24 h at 37 °C and 5% CO_2_. Previously, yeasts were incubated in the presence or absence of iDrk1 25 µg/mL for 24 h at 37 °C. After the interaction, each well was washed with sterile PBS (pH 7.2), coverslips were stained with hematology dyes (Newprov, Paraná, Brazil) and the supernatant was recovered for cytokine assay. The phagocytic index was determined from the protocol established by Popi et al. [36]. After 48 h, wells intended to quantify colony-forming units (CFU) were washed with 1 mL of ice-cold sterile ultrapure water and vigorously homogenized. The supernatant was plated in BHI medium supplemented with 10% FBS, and incubated for seven days at 37 °C. After growth, colony-forming units were counted. This experiment was carried out in biological triplicate.

The phagocytosis assay was also assessed by flow cytometry. Samples were prepared as described above. Prior to interaction, yeasts were labeled with CFSE (CellTrace™ CFSE Cell Proliferation—Thermo Fisher). For each 1 × 10^7^ Pb18 yeasts cells, 3 µL of CFSE (3 µg/µL) were added, with final volume of 500 µL of PBS (pH 7.2) and 0.5% BSA. Yeasts were incubated for 10 min at 37 °C, then centrifuged at 3000× *g* for 5 min and washed three times with PBS (pH 7.2). After 24 h, the samples were washed and the cells were detached from the wells using a cell scraper and homogenized in 500 µL of PBS (pH 7.2). Data acquisition was immediately performed in a flow cytometer (BD FACSCaliburTM, Becton Dickinson) using the FL-1 detection channel. After the first acquisition, 8 µL of Trypan Blue 0.4% was added to each sample to quench the signal of yeasts that could be adhered to the cell surface instead of internalized and again, the data was acquired. A total of 10,000 events were counted for each sample and the data were analyzed using the FlowJo software version 10.6.2 (FlowJo, LLC, FlowJo™, Ashland, OR, USA) [37].

### 2.11. Cytokines Determination

Supernatants from the phagocytosis assay were collected. The cytokines TNFα and IL12p70 were measured using the DuoSet ELISA kits (R&D Systems, Mineápolis, MN, EUA). The assay was performed in 96-well EIA/RIA plates according to the manufacturer’s specifications with modifications. First, plates were coated with 50 µL of capture antibody and incubated at room temperature for 16 h. Wells were blocked with 200 µL of diluent solution (1% BSA in PBS pH 7.2) and incubated at room temperature for 1 h. Then, 50 µL of culture supernatant were added, in triplicate, and 50 µL of the cytokine standard to perform the standard curve. Plates were then incubated for 2 h at room temperature. Next, 50 µL of Streptavidin-HRP solution (provided in the kit) were added to each well and the plate was incubated for 20 min protected from direct light. Finally, Tetramethylbenzidine (TBM) substrate was added and the plates were incubated for 20 min at room temperature, protected from light. At the end of the incubation, 50 µL of stop solution (2N H_2_SO_4_) was added. Between each incubation step, the wells were washed three times with 200 µL of wash buffer (0.05% Tween-20 in PBS pH 7.2). Absorbance was read at 430 nm (BioTek—Synergy HT).

### 2.12. Statistical Analysis

The data contained in this work were validated with the reproducibility of at least three independent experiments. For comparison analysis, a Student’s t-test and significance analysis were performed, as were the one-way variance (ANOVA), followed by Tukey’s test. Differences were considered significant when *p* < 0.05.

## 3. Results

### 3.1. Susceptibility of P. brasiliensis to Drk1 Pharmacological Inhibitors

**Figure 1:**
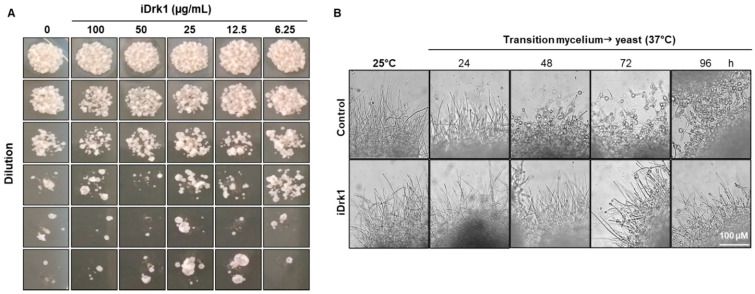
(**A**) Susceptibility of *P. brasiliensis* yeast cells to Drk1 pathway inhibitor (iDrk). A total of 1 × 10^6^ cells/mL were incubated with different concentrations of iDrk1 and incubated for 24 h. Then, the yeasts cells were diluted, plated in solid mYPD medium and incubated in for 7 days at 37 °C. (**B**) Dimorphic transition assay of *P. brasiliensis* in the presence or not of iDrk1. The fungus was cultivated as mycelial, at 25 °C and 150 rpm. Subsequently, it was plated in 6-well plates and incubated at 37 °C in mYPD medium supplemented or not with 20 µM iDrk1. Cultures were monitored every 24 h and observed under an optical microscope to evaluate the dimorphic transition.

Studies analysing the role of Drk1 in fungi [17,20] demonstrated that the use of specific inhibitors of group III histidine kinases (iprodione or fludioxonil) are efficient in promoting biological responses. Fludioxonil is a product derived from pyrrolnitrine, a compound isolated from *Pseudomonas pyrrocinia* [38]. The use of this inhibitor is already well established in studies with pathogenic fungi [17,39,40,41,42]. Initially, a susceptibility test was assessed with the Pb18 isolate. For this purpose, the yeasts were incubated for 24 h with different concentrations (100 to 6.25 µg/mL) of iDrk1 (fludioxonil) and then inoculated in mYPD medium. After 7 days of incubation, it was observed that there was no reduction in fungal viability at the concentrations tested (Figure 1A). Thus, based on the literature data [17], which demonstrated that a concentration of 25 µg/mL was able to inhibit the activity of Drk1 in *Blastomyces dermatitidis*, this concentration was established for the following experiments (Figure 1A).

### 3.2. Role of PbDrk1 in P. brasiliensis Cell Wall Maintenance

The transition from mycelium to yeast triggers the cell wall morphogenesis machinery, which involves synthesizing several cell wall sugars and proteins critical to survival during infection and evasion of the immune system [43]. In *Penicillium marneffei* pathogenic fungus, the role of *DRK1* is essential for stress adaptation, hyphal morphogenesis, and cell wall integrity [44]. Previously, we showed *PbDRK1* transcription to be phase-specific for the yeast form and demonstrated that PbDrk1 participates in dimorphic switching in *P. brasiliensis* when iprodione (another Drk1 inhibitor) is used [20]. To confirm the action of fludioxonil (iDrk1) in the *P. brasiliensis* yeast-mycelium switch, a dimorphic transition assay was performed. Initially, the fungus was cultivated in the mycelial form at 25 °C and then it was incubated at 37 °C in the presence or absence of iDrk1. The dimorphic transition was then followed every 24 h under an optical microscope. After 24 h at 37 °C, it was possible to observe the formation of yeasts in the distal portion of the hyphae in the control group, and at 96 h, there was a predominance of yeasts. In the group treated with iDrk1, at 96 h, there was a predominance of hyphae. Thus, the addition of fludioxonil also impairs the fungus’s ability to make the complete transition from mycelium to yeast (Figure 1B).

**Figure 2:**
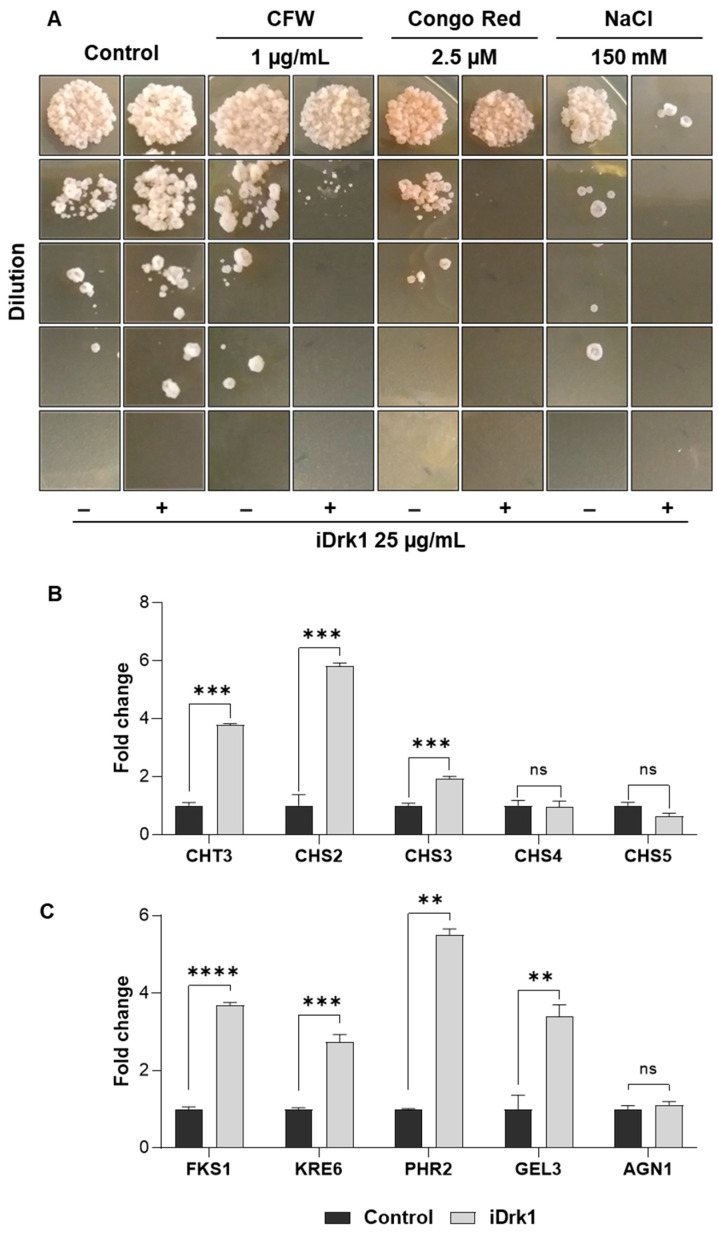
(**A**) Growth of *P. brasiliensis* in the presence of cell wall disrupting agents. A total of 1 × 10^6^ cells/mL were subjected to 25 µg/mL of iDrk1 and incubated for 24 h. Next, yeast cells were diluted, plated in solid mYPD medium containing different agents that disturb the cell wall, such as Calcofluor White (CFW), Congo Red and NaCl. Finally, cells were incubated in for seven days at 37 °C. Expression of cell wall morphogenesis related genes in *P. brasiliensis*. Pb18 cells were subjected to 25 µg/mL of iDrk1 for 24 h and then total RNA extraction was performed. The related genes (**B**) synthesis of chitin, such as *CHT3*, *CHS2*, *CHS3*, *CHS4* and *CHS5* and (**C**) synthesis of glucans such as *FKS*, *KRE6*, *PHR2*, *GEL3* and *AGN1* were analyzed, where *p*-value ≤ 0.01 (**), *p*-value ≤ 0.001 (***) and *p*-value ≤ 0.0001 (****).

In addition to the already known mechanism involving the dimorphic transition [17,18,20,45], it was observed that the strain deleted for Drk1 presents with sensitivity for conidia germination [44]. Based on this evidence, Pb18 yeasts were treated with iDrk1 and inoculated in YPDmod medium containing cell wall stressing agents such as Congo red (CR), Calcofluor White (CFW), and sodium chloride (Figure 2A). CR and CFW dyes are classically used in studies involving the synthesis and organization of fungal cell wall [46]. Both molecules have two groups of sulfonic acids that exert antifungal activity [47]. The action of CFW and CR occurs through binding to nascent chitin chains, preventing the access of enzymes that promote the binding of chitin with β-1-3-glucan and β-1-6-glucan chains. As a result, the cell wall becomes weakened, which can compromise its viability [47]. Sodium chloride promotes osmotic stress and changes the structure of the cell wall [48]. After seven days of incubation, it was observed that, under all conditions, previous exposure to iDrk1 induced a fairly dramatic viability reduction, especially under osmotic stress (Figure 2A). Previous data from our group [20] showed an increase in the number of *PbDRK1* transcripts when the fungus is subjected to osmotic stress. The data show that the inhibition of Drk1 results in an increased sensitivity to cell wall stressors and to osmotic stress.

### 3.3. Modulation of Cell Wall Gene Expression in P. brasiliensis

Previous data demonstrated that *PbDRK1* is mainly expressed in the yeast phase [20]. However, the associated pathways remain poorly understood. Therefore, to investigate the possible targets regulated by *PbDRK1*, Pb18 yeasts were incubated in the absence or presence of iDrk1 for 24 h. Then, the RNA extraction protocol was applied, followed by a cDNA synthesis reaction. Finally, RT-qPCR analysis of several genes involved in cell wall synthesis was performed: *CHT3*, *CHS2*, *CHS3*, *CHS4*, and *CHS5* (genes involved in the synthesis and maintenance of chitins) and *FKS1*, *KRE6*, *PHR2*, *GEL3*, and *AGN1* (genes involved in glucan synthesis and maintenance).

As shown in Figure 2B, genes encoding chitin synthase enzymes *CHT3*, *CHS2* and *CHS3* exhibited a 3.7-, 5.8- and 2.0-fold increase, respectively, in samples treated with iDrk1. However, there were no significant changes in the *CHS4* and *CHS5* genes (Figure 2B). On the other hand, the *CHS4* and *CHS5* genes were more highly expressed in the mycelial phase of Pb18 [49]. Thus, these data suggest that iDrk1 may modulate the expression of some chitin synthesis genes.

Significant increases in the transcript levels of several genes related to β-glucan synthesis and cell wall integrity have been observed (Figure 2C). A 4-fold increase in the *FKS1* gene, which encodes a β-(1,3)-glucan synthase, was observed. The *KRE6* gene showed a 2.7-fold increase in expression levels when compared with the no-treatment control. This gene is important in the β-(1,6)-glucan synthesis process and for molecules that are part of the β-(1,3)-glucan net composition [13,50]. The *PHR2* gene showed a 5.5-fold increase in expression levels (Figure 2C). This gene is involved in the maintenance of the cell wall and fungal virulence [51]. Finally, the expression of the *GEL3* gene exhibited a fourfold increase compared to the control without treatment. This gene is part of the process of elongation of the β-(1,3)-glucan chains and cell wall integrity [52,53]. On the other hand, the *AGN1* gene, which is involved in the maintenance and synthesis of α-(1,3)-glucan and highly expressed in the yeast phase [54], showed no difference in transcript levels (Figure 2C). These findings support the hypothesis that PbDrk1 could regulate negatively genes involved with the cell wall synthesis.

### 3.4. Modulation of the Cell Wall Components

As previously demonstrated, the inhibition of PbDrk1 was responsible for modulating the expression of genes involved in the synthesis of important cell wall components, such as *FKS1*. Associated with this, we observed a greater sensitivity of the fungus to cell wall disturbances when treated with iDrk1. Therefore, we decided to evaluate the levels of β-glucan, chitin and mannan in the Pb18 cell wall after treatment with iDrk1. For this purpose, β-glucan labelling was performed using the Dectin-1-Fc probe. Next, chitin levels were determined using the WGA (wheat germ agglutinin) marker conjugated with FITC [55]. This lectin molecule has an excellent affinity for N-acetyl-β-D-glucosaminyl residues and N-acetyl-β-D-glucosamine oligomers. Finally, to verify the mannan levels, concanavalin A conjugated with FITC was used [31]. This molecule has a high affinity for terminal residues of α-D-mannose and α-D-glucose.

**Figure 3:**
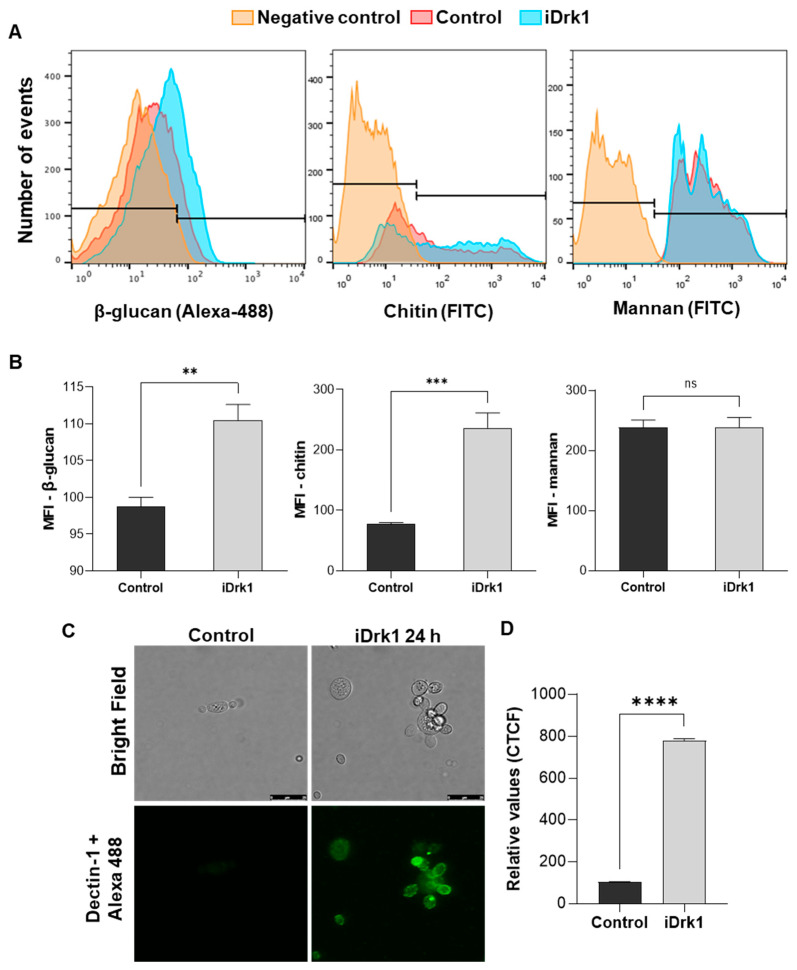
Quantification of *P. brasiliensis* cell wall components after incubation with iDrk1. Pb 18 cells were subjected to 25 µg/mL of iDrk1 for 24 h and then labeled with fc-Dectin-1 and Alexa 488 for dosage of β-glucan, WSA conjugated with FITC for dosage of chitin oligomers and Concanavalin A conjugated with FITC for mannan dosage. (**A**) Histograms from each cell wall component by flow cytometry, where negative control is represented as yeast that were not fluorescence labelled. (**B**) Quantification through the median fluorescence intensity (MFI) where *p*-value ≤ 0.004 (**). Evaluation of β-glucan exposure in *P. brasiliensis* cell wall after incubation with iDrk1. Cells of Pb18 were subjected to 25 µg/mL of iDrk1 for 24 h and then labeled with fc-Dectin-1 and Alexa 488. (**C**) Confocal microscopy analysis. (**D**) Corrected quantification of total fluorescence (CTCF) from confocal microscopy analysis where *p*-value ≤ 0.01 (**), *p*-value ≤ 0.001 (***) and *p*-value ≤ 0.0001 (****).

Yeasts were incubated for 24 h in the presence or absence of iDrk1. Then, labelling was performed for each fluorescent marker described above. Fluorescence analyses were obtained through flow cytometry, where 10,000 events were obtained for each sample. Quantification was performed using the median fluorescence intensity (MFI). Figure 3A,B show a significant increase in β-glucan and chitin levels after 24 h of treatment with iDrk compared to the untreated control. On the other hand, no change in mannan levels was observed. In addition to the flow cytometry measurements, β-glucan localization was also evaluated by confocal microscopy. Yeast labelling was performed as described in the previous section using the Dectin-1-Fc probe and a secondary antibody anti-mouse IgG conjugated to Alexa 488. This assay made it possible to observe an increase in labelling in the fungus previously incubated with iDrk1 compared to the control. Most of the marking was observed on the yeast surface (Figure 3C). Fluorescence quantification (Figure 3D) was obtained from the corrected total fluorescence values (CTCF). These results indicate that PbDrk inhibition increases the β-glucan levels in *P. brasiliensis* yeast cells.

### 3.5. Inhibition of PbDrk1 Induces Increased Phagocytosis of P. brasiliensis and Alters Cytokine Production by Macrophages

**Figure 4:**
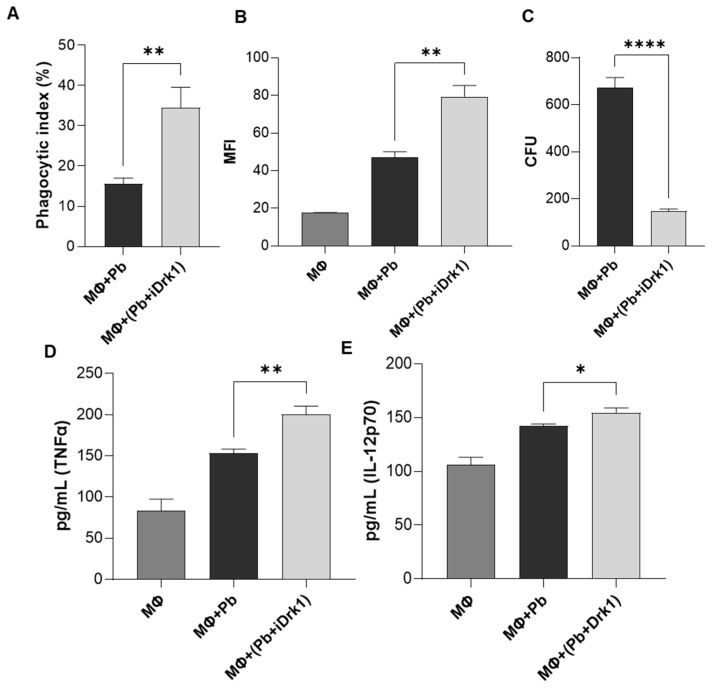
Phagocytosis assay. 2.5 × 10^5^ J774 cells (MΦ) were plated in RPMI medium supplemented with 10% FBS. Then the cells were primed for 30 min with LPS 100 ng/mL. In parallel, *P. brasiliensis* yeasts were subjected to 25 µg/mL of iDrk1 for 24 h. The interaction was carried out in a 2:1 ratio (yeast:macrophages) for 24 h at 37 °C and 5% CO_2_. After this period, the cells were stained and visualized under an optical microscope to count the internalized yeasts. (**A**) Phagocytic index was calculated, where *p*-value ≤ 0.005 (**). (**B**) Phagocytosis assay performed by labeling *P. brasiliensis* yeasts with CFSE prior to interaction with macrophages. Fluorescence quantification was performed by flow cytometry and relative quantification was obtained through the median intensity (MFI) where *p*-value ≤ 0.005 (**). (**C**) CFU determination from yeasts recovered from macrophages, where *p*-value ≤ 0.0001 (****). Cytokine assay from J774 cell supernatant after interaction with *P. brasiliensis* submitted or not with iDrk1. Macrophages were plated in RPMI medium supplemented with 10% FCS. Then, cells were primed for 30 min with LPS 100 ng/mL. In parallel, *P. brasiliensis* yeasts were subjected to 25 µg/mL of iDrk1 for 24 h. The interaction was carried out in a 2:1 ratio (yeast:macrophages) for 24 h at 37 °C and 5% CO_2_. After this period, the supernatant was collected and the cytokines (**D**) TNFα, (**E**) IL-12p70 were measured, where *p*-value ≤ 0.05 (*), *p*-value ≤ 0.01 (**).

Fungal cell wall β-glucan is also an important pathogen-associated molecular pattern (PAMP). To avoid the innate immune response, many fungal pathogens depend on the synthesis of the cell wall α-glucan, which functions as a stealth molecule to mask the β-glucans itself or links other masking structures to the cell wall [56]. As demonstrated in the previous results, when we inhibited the PbDrk1 pathway, the Pb18 yeast underwent changes in their cell wall composition. Under normal conditions, the main components of the outermost layer of the Pb18 cell wall are α-glucan molecules [13,57]. Thus, we determined whether the increase in β-glucan would make the fungus more susceptible to recognition by cells of the immune system. To answer this question, a phagocytosis assay was performed with J774 murine macrophages. After 24 h of interaction, the supernatant was collected for subsequent cytokine dosage. In this assay, it was possible to observe that fungi treated with iDrk1 had a higher phagocytic index than the untreated control (Figure 4A). To confirm these observations, a phagocytosis assay was performed using flow cytometry [37]. The sample treatment was performed as described above, but before the cell-yeast interaction, the Pb18 yeasts were labelled with the intracellular dye CFSE. This result is similar to that observed in the previous experiment. In Figure 4B, we can see the greater signal intensity in the sample where the fungus was previously treated with iDrk1. Furthermore, we observed a reduced number of colony-forming units (CFUs) in the group treated with iDrk1 (Figure 4C). This dataset suggests that PbDrk1 inhibition of *P. brasiliensis* may have contributed to an increase in phagocytosis and in the susceptibility of fungal cells to macrophage elimination.

The quantification of the proinflammatory cytokines TNFα and IL-12p70 was evaluated by ELISA. Before the phagocytosis assay, J774 cells were primed with LPS. As a control, the supernatant from the macrophages activated only with LPS was analysed. In Figure 4D, we can see a significant increase in TNFα levels in the supernatant of cell cultures incubated with *P. brasiliensis* yeasts previously exposed to iDrk1. The same result was observed for the cytokine IL-12p70 (Figure 4E).

### 3.6. Regulation of cAMP-PKA and Glycogen Accumulation in P. brasiliensis

**Figure 5:**
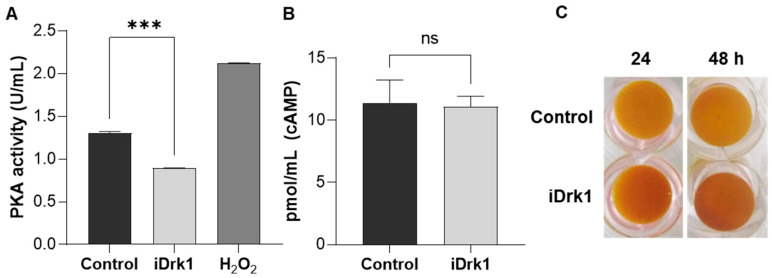
PKA activity and cAMP quantification in *P. brasiliensis* after incubation with iDrk1 (**A**) Quantification of PKA activity and (**B**) dosage of *P. brasiliensis* cAMP after 24 h incubation with 25 µg/mL of iDrk1, where *p*-value ≤ 0.001 (***). (**C**) After 24 and 48 h of yeast incubation in the presence of the inhibitor, the cells were stained with a 0.2% iodine and 0.4% potassium iodide solution to assess glycogen accumulation.

Perception of the environment is fundamental for fungal survival in the host. The cyclic AMP-dependent protein kinase A pathway (cAMP-PKA) is highly conserved and is involved in several biological processes, both in human pathogenic and phytopathogenic fungi [58]. In addition, it also contributes to gene expression regulation and cell wall remodeling [59]. Through stimuli external to the cell, the enzyme adenylate cyclase converts ATP to adenosine 3′,5′-cyclic monophosphate (cAMP), an important secondary messenger that binds to the catalytic subunit of protein kinase A (PKA). This generates a conformational change that releases PKA catalytic subunits and activates transcription factors and other signalling pathways involved in cell wall integrity, stress response and virulence [58,60,61]. Thus, Pb18 yeasts treated or not with iDrk1 for 24 h were lysed, and the protein extract was used to quantify the PKA activity and cAMP dosage. As shown in Figure 5A, it was possible to verify a reduction in PKA activity after exposure to iDrk1. As a positive control for the reaction, *P. brasiliensis* was subjected to oxidative stress with 300 mM hydrogen peroxide (H_2_O_2_) for 30 min. This condition is known to generate an increase in PKA activity [62]. It was also possible to observe no significant difference in the dosage of cAMP levels between the treated and control samples (Figure 5B).

The cell stress response is orchestrated by connecting several pathways that converge to promote cell survival. As previously described, the cell wall integrity pathway also activates responses that bind to cAMP-PKA. Activation of PKA regulates cellular functions related to glycogen metabolism [63], and it antagonizes its intracellular accumulation [64]. Thus, Pb18 yeasts incubated in the presence or absence of iDrk1 for 24 and 48 h were stained with iodine solution and photographed (Figure 5C). The greater the accumulation of intracellular glycogen, the darker the sample becomes [33]. In Figure 5C, there was an accumulation of intracellular glycogen in relation to the control. Combined with the gene expression analysis and quantification of the cell wall components, this result suggests that iDrk1 may modulate the cell wall component synthesis, generating a cellular response that leads to a decrease in PKA activity. This decreased activity may lead to intracellular glycogen accumulation [65,66].

## 4. Discussion

Currently, the number of antifungal substances available for the treatment of phytopathogens is approximately nine times greater than those available for the treatment of mycoses in mammals [67]. In this scenario, it is important to emphasize the need for studies that unravel the mechanisms of fungal pathogenicity. With a better understanding of these pathways and the discovery of new targets, it will be possible to develop new drugs with antifungal potential. Thus, this study aimed to understand better the role of a histidine kinase (PbDrk1), an important regulator in the dimorphic switch and morphogenesis of the cell wall of *P. brasiliensis*.

We evaluated pharmacological inhibitors as a strategy to elucidate the role of PbDrk1. The inhibitors iprodione and fludioxonil (both group III histidine kinase inhibitors) are substances widely used in agriculture to combat phytopathogens [68]. Molecular biology and cell signalling studies have shown that these molecules act specifically on group III histidine kinases through interactions with HAMP domains, which are present only in this group of kinases [39,69]. In *Saccharomyces cerevisiae*, group III histidine kinases are absent. When an orthologous gene to histidine kinase group III of *Neurospora crassa* is introduced, *S. cerevisiae* becomes sensitive to pyrrolnitrine [68,70]. Other pathogenic fungi with group III histidine kinases, such as *B. dermatitidis* and *Candida albicans*, were submitted to this inhibitor and presented various sensitivities [17]. For Pb18 yeast cells, fludioxonil was used at 100 µg/mL, showing no reduced cell viability. Thus, the concentration established in the other tests at 25 µg/mL was selected since it is an intermediate concentration and was already established in *B. dermatitidis* [17].

The fungal cell wall plays a fundamental role in the host-parasite interaction since its composition can influence the immune response [13]. In *Paracoccidioides* spp., during the dimorphic transition, the cell wall morphogenesis machinery is activated to remodel its components. In *H. capsulatum* and *B. dermatitidis,* it was shown that *DRK1* acts as a regulator of dimorphism and virulence. A study carried out in *B. dermatitidis* silenced the *DRK1* gene and demonstrated a blockade of the dimorphic transition from mycelium to yeast at 37 °C. Furthermore, this silencing impaired the expression of *BAD1* (a virulence gene activated during transition) [11]. In *H. capsulatum*, Drk1 regulates genes specifically expressed in the yeast phase, such as *CBP1*, *YPS-3* and *AGS1* [11]. In *C. albicans*, the deletion of *NIK1*, a group III histidine kinase, makes yeast incapable of transitioning from yeast to hyphae, consequently making it less virulent [71]. In *Penicillium marneffei*, the deletion of the *DRKA* and *SLNA* genes was also essential for the dimorphic transition during macrophage infection and conidial germination, respectively [44].

The ability of *Paracoccidioides* spp. to cause PCM depends on its dimorphic transition and establishment in the host, either by the resistance and evasion of the immune response machinery or by its ability to be a facultative intracellular parasite [8]. The use of iDrk1 (fludioxonil) in *P. brasiliensis* cultivated in the form of mycelium prevented it from performing the dimorphic transition efficiently, confirming previous findings [20]. A study carried out in *Penicillium marneffei* showed the importance of *DRK1* in cell wall morphogenesis. Strains deleted for this gene were not able to grow in a medium supplemented with cell wall stressors (Congo red) and osmotic stress agents (NaCl and sorbitol) [44]. In that same study, *DRK1* mutant hyphal growth and conidial germination were affected, and transmission electron microscopy images showed significant thickening of the cell wall [44]. These data support the results obtained in Pb18. In this work, yeasts submitted to iDrk1 and later inoculated in culture medium supplemented with cell wall stressors had severely impaired growth.

In *Paracoccidioides* spp. the cell wall chitin content represents a significant fraction of the cell dry weight [49]. The *CHT3*, *CHS2,* and *CHS3* genes showed significant increases compared with the control that was not treated with iDrk1. In *C. albicans*, it was observed that overexpression of *CHT3* induces greater sensitivity to cell wall stressors, and the *CHS2* gene acts as an important regulator [72,73]. In *P. brasiliensis*, it is known that the *CHS3* gene is mainly expressed in the yeast phase [49] and is related to cell growth [74]. These results suggest that PbDrk1 inactivity may modulate the expression of some genes involved in chitin synthesis, leading to cell wall instability.

In addition to chitin, one of the main elements that comprise the cell wall of *Paracoccidioides* spp. are glucan molecules. As already mentioned, the predominance of β-glucan during the mycelial phase is reverted to α-glucan in the yeast phase [2]. This mechanism is seen as one strategy to evade the immune system. The level of α-glucan in the cell wall can be related to the degree of virulence [14]. Among the genes analysed, *AGN1* was characterized as phase-specific in *P. brasiliensis* yeasts, regulating the synthesis of α-(1,3)-glucan [54]. Our data showed that PbDrk1 does not participate in the modulation of this gene. On the other hand, genes involved in the synthesis and maintenance of β-(1,3)-glucan and β-(1,6)-glucan chains, such as *FKS1*, *KRE6*, *PHR2*, and *GEL3*, showed significant increases after exposure to iDrk1. These results indicate that PbDrk1 regulates the expression of cell wall synthesis genes directly related to the dimorphic transition. The expression data were confirmed by evaluating the levels of β-glucan and chitin in Pb18 treated with a Drk1 inhibitor. The glucan modulation transition is essential for masking β-glucan molecules, as the host’s immune system directly recognizes these molecules by phagocytic cells.

In the host, the fungus is initially recognized by PRRs and PAMPs [75]. Among the receptors that may be involved, we highlight the Dectin-1 receptor that specifically binds to β-glucan [76]. Chitin molecules are also recognized by the immune system via TLR-2, inducing the production of cytokines and the recruitment of phagocytic cells by the recognition by Dectin-1 [77]. The outermost region of the *P. brasiliensis* cell wall comprises a thick layer of mannan [2]. This layer is believed to protect fungal cells from immunological recognition, hiding the main immunogenic molecules [78]. Confocal microscopy assays indicated an increase in β-glucan labelling. These images showed that yeasts treated with iDrk1 showed greater fluorescence intensity when labelled with the Dectin-1 receptor. Thus, the increase in β-glucan and increased expression and synthesis of chitin in cells treated with iDrk1 indicate that this pathway may be related to virulence and fungal cell wall architecture.

In parallel, the cAMP-PKA pathway also plays a role in cell wall remodeling. In *Cryptococcus neoformans*, PKA is involved in the phosphorylation of components that, when translocated to the nucleus, regulate the expression of genes involved in cell wall integrity [59]. In fungi, this pathway is related to cell growth, differentiation, stress response, pathogenicity, and cell wall integrity, among others [61]. In *C. neoformans,* it was shown that the deletion of several genes involved in cell wall integrity led to impairment of the cAMP-PKA pathway [79]. In *C. albicans*, this pathway plays a central regulator of its morphological transition and, consequently, pathogenicity [80]. In *P. lutzii,* it was reported that the specific inhibition of PKA impedes dimorphic transition [81]. Recently, Garcia et al.; (2017) showed a new insight into the signalling pathways involved in the regulation of cell wall integrity. Alterations of the β-1,3-glucan network in the fungal cell wall induced the activation of the CWI pathway and in parallel inhibited the PKA signalling [33]. Thus, when submitting Pb18 yeasts to iDrk1, we observed a reduction in PKA activity, supporting literature data on the PKA influence on cell wall gene expression and glycogen accumulation [33,59] and a possible correlation with the PbDrk1 pathway.

On the other hand, there was no significant difference in cAMP levels in the presence or absence of iDrk1. Cyclic AMP is a secondary messenger whose intracellular levels are regulated by adenylate cyclase and the phosphodiesterase enzyme balance [65]. Both are regulated by other signalling pathways and not exclusively via PKA [82]. Thus, it is possible to infer that PbDrk1 modulates PKA activity but does not correlate with cAMP levels. In this case, the maintenance of cAMP levels can be regulated by other pathways, such as calcineurin, MAP kinases, and G protein subunits [80,83]. Finally, we can infer that PbDrk1 modulates intracellular levels of intracellular glycogen via PKA. In *Aspergillus fumigatus*, deletion of the PKA catalytic subunit led to an increase in intracellular glycogen levels [65]. Furthermore, in *S. cerevisiae*, it was found that glycogen is present in two fractions, one of them in the cell cytosol in its soluble form and the other associated with cell wall components, covalently linked specifically to β-(1,3)-glucan and β-(1,6)-glucan [84]. This evidence complements the observed results of increased β-glucan exposure on the surface of *P. brasiliensis* after exposure to iDrk1. Together, the results obtained thus far point to the participation of PbDrk1 in the *P. brasiliensis* cell wall modulation.

During the immune response in a fungal infection, the action of pro- and anti-inflammatory cytokines is essential to determine disease progression and/or pathogen clearance [85]. Host resistance to infection by *Paracoccidioides* spp. is associated with a Th1 response, which induces macrophage activation and actively controls fungal dissemination [86]. The individual’s susceptibility to the disease is associated with a Th2 response [87]. It is known that the participation of the Dectin-1 receptor together with TLR in the recognition pathway of *Paracoccidioides* spp. triggers the production of several proinflammatory cytokines [88], such as TNFα. This molecule is produced by cells involved in the immune system, including activated macrophages and regulatory T cells, acting as central mediators in inflammation and the regulation of the immune response [89]. It is possible to observe the increased production of this cytokine by macrophages that phagocytosed yeasts exposed to iDrk1, indicating a more exacerbated proinflammatory response when compared to the control. Furthermore, a significant increase in the expression of the cytokine IL12p70 was also observed, produced by monocytes, macrophages, and dendritic cells, and it is an important element in the activation of the Th1 response [90].

## 5. Conclusions

The data obtained during this work will contribute to a better understanding of a regulatory pathway that has not yet been explored in this model. The PbDrk1 protein has already been shown to be a key element in the dimorphic transition pathway [20]. It is now possible to state that it is a likely virulence factor in regulating cell wall genes. The activity of PbDrk1 negatively modulates the synthesis of molecules such as chitin and β-glucans, contributing to its masking and favouring the pathogenicity of *P. brasiliensis*. Histidine kinases are proteins absent in mammals, and the inhibition of their activity makes fungal cells susceptible to immune system cells. In other fungal models, the loss of this gene represents a decrease in virulence. Finally, the study of PbDrk1 and associated pathways would enable a different approach to the development of new antifungal drugs.

## Figures and Tables

**Table 1:** Oligonucleotides used for real-time quantitative PCR analysis.

Gene	Sequence (5′–3′)	Gene ID
*L34*	Foward: AAAGGAACCGCACCAAAATG Reverse: AGACCTGGGAGTATTCACGG	PADG_04402
*18S*	Foward: CGGAGAGAGGGAGCCTGAGAA Reverse: GGGATTGGGTAATTTGCGC	ADG_12090
*FKS1*	Forward: GTTCCCATCACCGATCCTATTT Reverse: GAAGGAGAGCAAGAAGACGATAC	PADG_11846
*KRE6*	Foward: TTCCGACGAGTTCAACAAAGA Reverse: CTGCGTCACTCCATACCAAATA	PADG_07170
*PHR2*	Foward: ACTGAGGACAAACACCATCAG Reverse: ACAGATCTGCAACGACGTAAA	PADG_04918
*GEL3*	Foward: CGTTGTCAGCGGAGGTATCGTC Reverse: AGGGCAGGTTCGGAGTTCAGTG	PADG_04918
*AGN1*	Foward: AAATGCGGCACGGAGGAGA Reverse: AAGGGTGGTATCAAGTGCCGAGT	PADG_03169
*CHT3*	Foward: GCGAGGAATTGGGTGATAGAA Reverse: AGGGTTGACGCTATCAGAAATAA	PADG_08156
*CHS2*	Foward: CCCGAACCTACTGCACTTTATC Reverse: TGCCCTTACCCGCTTTAATC	PADG_08636
*CHS3*	Foward: CGCTATGGTTAAGGATCCCGAGA Reverse: GCATCCAGGCAAGCAAGTAACA	O94191_PARB
*CHS4*	Foward: ACCGGATGAGGCCACTATTACAGA Reverse: GTCTGCAATCGCTGCTCAACG	PADG_07911
*CHS5*	Foward: AGAGTATCAAGGCTGAGCTGGAACG Reverse: CGGAAAGGACGGCTTCGGTT	A9XTF9_PARBR
